# Online Classification of Contaminants Based on Multi-Classification Support Vector Machine Using Conventional Water Quality Sensors

**DOI:** 10.3390/s17030581

**Published:** 2017-03-13

**Authors:** Pingjie Huang, Yu Jin, Dibo Hou, Jie Yu, Dezhan Tu, Yitong Cao, Guangxin Zhang

**Affiliations:** State Key Laboratory of Industrial Control Technology, College of Control Science and Engineering, Zhejiang University, Hangzhou 310027, China; huangpingjie@zju.edu.cn (P.H.); 21432051@zju.edu.cn (Y.J.); yu_jie@zju.edu.cn (J.Y.); 21632031@zju.edu.cn (D.T.); 130910215@mail.dhu.edu.cn (Y.C.); gxzhang@zju.edu.cn (G.Z.)

**Keywords:** early warning systems, contaminant classification, conventional water quality sensors, support vector machine, multi-classification probability output

## Abstract

Water quality early warning system is mainly used to detect deliberate or accidental water pollution events in water distribution systems. Identifying the types of pollutants is necessary after detecting the presence of pollutants to provide warning information about pollutant characteristics and emergency solutions. Thus, a real-time contaminant classification methodology, which uses the multi-classification support vector machine (SVM), is proposed in this study to obtain the probability for contaminants belonging to a category. The SVM-based model selected samples with indistinct feature, which were mostly low-concentration samples as the support vectors, thereby reducing the influence of the concentration of contaminants in the building process of a pattern library. The new sample points were classified into corresponding regions after constructing the classification boundaries with the support vector. Experimental results show that the multi-classification SVM-based approach is less affected by the concentration of contaminants when establishing a pattern library compared with the cosine distance classification method. Moreover, the proposed approach avoids making a single decision when classification features are unclear in the initial phase of injecting contaminants.

## 1. Introduction

Water supply systems are vulnerable to chemical and biological contamination because of interferences in the external environment. Therefore, an early warning system (EWS) for water quality should be established as an effective measure in mitigating the effects of pollution [1,2]. Quickly detecting the presence of contaminants and identifying the type of contaminants, as well as providing help for implementing remedial measures are the EWS core competencies.

The subsequent key problem after detecting the pollutants in EWS is identifying the types of pollutants. The laboratory-based analysis method is a common method for classifying pollutants (e.g., ICP-MS). This analysis method includes accurate detection and quantification; however, it has long detection time. Time is a critical factor for emergency water pollution [3,4]. Therefore, developing a rapid method for classifying pollutants is urgent. The use of online compound specific sensors for detection is a viable method; this method is faster than the laboratory-based analysis. However, a specific composite sensor can only identify a single or a small class of pollutants. This limitation will inevitably lead to inefficiency and recognition failure.

To overcome this limitation, some researchers have attempted to build a real-time classification method of pollutants using conventional indicators. Kroll [5] reported the Hach Homeland Security Technologies (HST) approach that processes a plurality of independent quality parameters into a single deviation signal to detect an abnormal water quality compared with a preset threshold. Simultaneously, the direction of the deviation signal vector represents that the characteristics of the agents can be used to classify pollutants further. Yang et al. [6] conducted a series of experiments based on a pilot experiment system and reported a new real-time adaptive method for contaminant classification. In this method, four discriminative systems were established according to the response of different conventional indicators caused by 11 different pollutants, which were divided into three categories: fast reaction, slow reaction, and no reaction. In the two methods, the direction of the deviation signal vector and the geometric properties of the pollutant curve were used to distinguish the pollutants. The classification of pollutants is based on qualitative analysis rather than quantitative analysis. However, the qualitative analysis method may fail when the pollutants have a similar response. Therefore, classifying pollutants based on quantitative analysis is necessary. Liu et al. [7] used a Mahalanobis distance measure similarity between pollutant characteristic vector, thereby calculating the Mahalanobis distance between the pollutant feature vector and the various pollutant feature vectors in the feature vector library. Then, the category of pollutant can be determined as a type of class having the minimum distance. Later, Liu et al. [8] used cosine distance measure similarity between pollutant characteristic vectors. Compared with the Mahalanobis distance, the cosine distance is only related to the direction of feature vectors, where the various components of the feature vector change simultaneously. This change can reduce the impact of the concentration of pollutants, resulting in the magnitude change of the feature vector. However, in constructing the pollutant characteristic libraries, the cosine distance classification methodology uses the mean of all of the instances in same class with different concentrations. This method can lead to some deviations when calculating the similarity between the new samples and the features in the pattern library.

The support vector machine (SVM) is a classification method based on statistical learning theory. SVMs effectively solve the problems of having a small sample size, high dimension, and nonlinearity in the learning process, and remarkably improve the generalization ability of the learning method. SVMs aim to find an optimal hyperplane that maximizes the margin between classes by using a small number of training cases, namely, the support vectors, which have distinct features in some instances. Currently, SVMs are widely used in handwriting recognition, face recognition, fault classification [9,10,11], time series prediction [12], nonlinear system modeling and identification [13,14], water quality event detection [15] and other fields. The use of SVMs has attained good results.

For the above limitations, this study proposes a classification method for pollutants based on multi-classification probability output (MCPO). This method extends a binary SVM to a multi-classification SVM and introduces a probability output. The proposed method reduces the influence of the concentration of pollutants in the process of building a pattern library. Moreover, the proposed method can identify whether the feature of the sample is readily apparent. The samples, which classification characteristics are readily apparent, can determine their category; then, the samples, which classification characteristics are not apparent, avoid making a single classification decision.

## 2. Methodology

**Figure 1:**
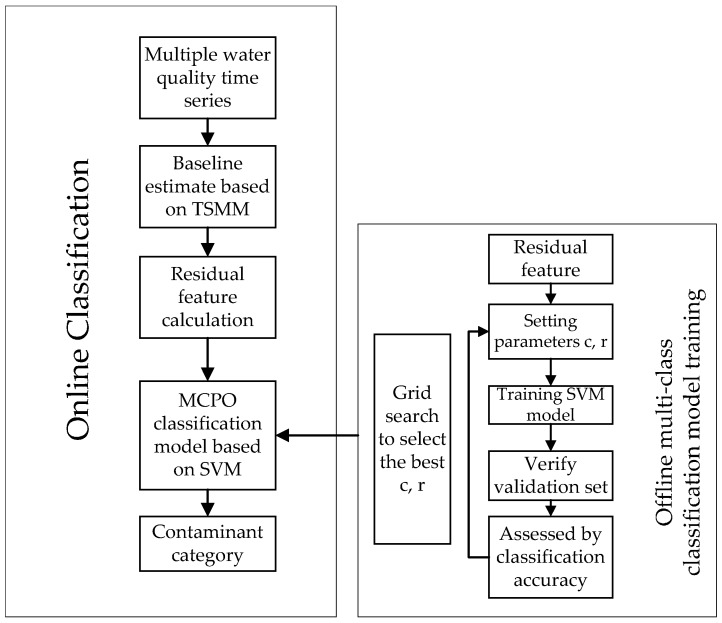
Flowchart of pollutants on-line classification.

This study proposes a novel method that work in two steps, as shown in Figure 1. First, an offline SVM-based MCPO classifier is trained. Second, an online classification framework is employed to obtain the output of the SVM-based MCPO model for further analysis, consequently arriving at the precise classification of pollutants.

### 2.1. Baseline Estimate Based on Time Series Movement Mean (TSMM)

The TSMM represents a hidden predicting model, the average of a period of time before the water quality parameter is used as current predicted value $Z^{*}(t)$.

(1) $$Z^{*}(t)\;=\;\frac{1}{n}\sum_{i=1}^{n}Z(t-i),$$

Residual δ(t) series are obtained by using the average of normal indicators of water quality within a sliding time window as the background signal of water quality. The residual value is used as a characteristic in the pollutant anomaly detection and classification [16,17,18], which can significantly reduce the influence of fluctuations in the water quality background.

(2) $$\delta (t)\;=\;Z(t)-Z^{*}(t)=Z(t)-\frac{1}{n}\sum_{i=1}^{n}Z(t-i),$$

The residual value exceeding the threshold can be considered an outlier, and the outlier does not have to be added to the calculation of the background signal of water quality. When the number of continuous outliers exceeds the upper limit, the background data of water quality are considered altered.

### 2.2. MCPO Classification Model Based on SVM

#### 2.2.1. Fundamentals of SVM

SVM theory was suggested by Vapnik et al. [19,20] in statistic learning theory. The basic principle is using a hyperplane determined by a number of support vectors to classify data. The support vector is a subset of training set and is used for determining a decision boundary of all types of data.

In classifying water pollution, the conventional indicators of the low concentrations of pollutants characteristic response, including inconspicuous error-prone points, constitute a subset of the support vector of SVM, thereby determining the dividing hyperplane between classes.

The SVM developed from an optimal classification hyperplane in the case of linear separable. The linear separable training set, $\left(x_{i},y_{i}),i=1,...,n,x\in R^{d},y\in \{+1,-1\right\}$, satisfies

(3) $$y_{i}[(w\cdot x_{i})+b]-1\geq 0,i=1,...,n,$$

The classification plane, namely, the optimal classification hyperplane, results in the maximum classification margin. This result is required for the promotion of capacity control as the maximum classification margin, which is one of the core concepts of SVM. The above optimal classification problem can be transformed into a dual problem through Lagrange optimization method. The above problem can be solved by obtaining the optimal classification function as:

(4) $$f(x)=\mathrm{sgn}\{(w\cdot x)+b\}=\mathrm{sgn}\{\sum \alpha_{i}^{*}y_{i}(x_{i}\cdot x)+b^{*}\},$$

Only a part (usually a few) of the above solution $\alpha_{i}$ will not be zero. The summation is applied to the support vector. $b^{*}$ is the classification threshold.

The nonlinear problem can be converted to a high-dimensional space through a nonlinear transformation and thus seek an optimal classification surface in the transformation space. A proper kernel function $k(x_{i},x_{j})$ is adopted in the optimal classification hyperplane, which leads to a linear classification to be achieved after the non-linear transformation. The computational complexity has not increased, thus the corresponding classification function becomes

(5) $$f(x)=\mathrm{sgn}\{\sum \alpha_{i}^{*}y_{i}K(x_{i}\cdot x)+b^{*}\},$$

A different inner product kernel function will form different algorithms in the SVM. Four types of kernel functions are widely applied.

1. Linear kernel function: K(x,y)=x⋅y;
2. Polynomial kernel function: $K(x,y)={\left[(\lambda \cdot x\cdot y)+\alpha \right]}^{d}$;
3. Radial basic function: $K(x,y)=\exp\{\frac{||x-y|{|}^{2}}{\sigma^{2}}\}$; and
4. Sigmoid function: K(x,y)=tanh(ν(x,y)+c).

The SVM model proposed above is derived by single classification results. Misclassification is common when the conventional indicator response characteristics are unclear at the beginning of pollutant injection. Therefore, probability output is proposed to obtain the classification probabilities of pollutants, thereby avoiding the creation of a single decision. Currently, numerous researchers have proposed various programs to develop a two-class SVM output probability value [21]. In this study, the widely used method of Platt [22] is adopted; this method uses a sigmoid function, including parameters, maps an SVM decision function into the interval [0, 1], and then achieves the probability output as follows:

(6) $$p(y=1|x)\approx p_{A,B}(f)=\frac{1}{1+\exp(Af+B)},$$

where f=f(x) denotes the decision function. A and B are the undecided parameter of the sigmoid function, and A < 0 ensures that the sigmoid function is an increasing monotone. A and B can be obtained by applying the training set $\left(f_{i},y_{i}\right)$ with the maximum likelihood estimation.

#### 2.2.2. Multi-Classification Probability Based on SVM

Classifying pollutants is a multi-classification problem. In this study, the one-against-one method is used to extend the binary SVM model to multi-classification. In this case, the integrated k(k−1)/2 two-class SVM classification results, and the probability estimation $r_{ij}$ are synthesized as the MCPO, and the probability $P_{i}$ belonging to each class is obtained, $p_{i}=P(y=i|x),i=1,2,...,k$, $\sum_{i=1}^{k}p_{i}=1$.

In this study, the method of multi-class probability comes from the two-class probability suggested by Wu [23,24]. Given that $r_{ij}+r_{ji}=1$, $r_{ij}\approx p_{i}/(p_{i}+p_{j})$ and $r_{ij}/r_{ji}\approx p_{i}/p_{j}$, the simple conversion and summation is as follows:

(7) $$\sum_{j\neq i}r_{ji}p_{i}\approx \sum_{j\neq i}r_{ij}p_{j},$$

Using the relationship between $r_{ij}$ and $P_{i}$ in Equation (7), $P_{i}$ can be obtained by dealing with the following model:

(8) $$\left\{ \begin{array}{l} \min\sum_{i=1}^{k}\sum_{j\neq i}{\left(r_{ji}p_{i}-r_{ij}p_{j}\right)}^{2}\\ s.t.\;\sum_{i=1}^{k}p_{i}=1\;p_{i}\geq 0 \end{array}\right.,$$

where the constraint $p_{i}\geq 0$ is redundant, denoting $p^{T}=[p_{1},p_{1},...,p_{k}]$. The model is transformed into Equation (9), which is a convex quadratic programming problem. When Equation (10) is satisfied, the optimal solution is obtained.

(9) $$\left\{ \begin{array}{l} \underset{p}{\min}2p^{T}Qp=\underset{p}{\min}\frac{1}{2}p^{T}Qp\\ Q_{ij}=\left\{ \begin{array}{ll} \sum_{s\neq i}r_{si}^{2} & i=j\\ -r_{ji}r_{ij} & i\neq j \end{array}\right. \end{array}\right.,$$

(10) $$\left[\begin{array}{ll} Q & e\\ e^{T} & 0 \end{array}\right]\left[\begin{array}{l} p\\ b \end{array}\right]=\left[\begin{array}{l} 0\\ 1 \end{array}\right],$$

where $e\;=\;{\left[1,1,...,1\right]}^{T}$.

Iteratively, let t=1,...k,1...k,1.... Then, the process of solving the optimal solution is as follows:

1. Utilize Equation (11) to update $p_{t}$
  (11) $$p_{t}\leftarrow \frac{1}{Q_{tt}}(-\sum_{j\neq t}Q_{tj}p_{j}+p^{T}Qp),$$
2. Normalize the parameter p.
3. Verify whether p satisfies Equation (9); if satisfied, then stop the iteration, and obtain the multiple classification probabilities p.

### 2.3. Parameter Selection of SVM Model

The effectiveness of SVM depends on selecting a kernel function and the penalty parameter C. The radial basis function is usually selected as a kernel function.

**Figure 2:**
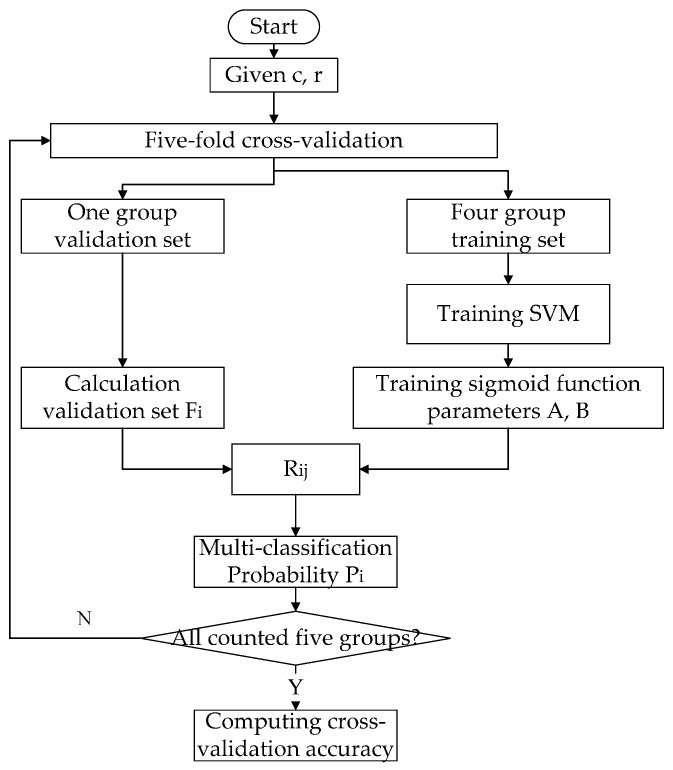
Flow-chart of the calculation process in selecting the parameters for the SVM model.

Typically, the parameter γ in the kernel function and penalty parameter C are checked using cross-validation. In this study, a grid search method is utilized for the training set through cross-validation, selecting a group (γ, C) of the highest classification accuracy as a model parameter. The penalty parameter C is in the range of 0.01, 0.1, 1, 10, and 100, while nine values exist for each interval, with a total of 37 values. The parameter of the radial basis function has a range of 0.01, 0.1, 1, 10, and 100, while each interval has nine values, with a total of 37 values. Consequently, a total of 1369 choices exist. Figure 2 shows the flow-chart of the calculation of the parameter accuracy rate.

### 2.4. Evaluation of Classification Performance

#### 2.4.1. Confusion Matrix

A confusion matrix is primarily used to compare the classification results with the actual measured values. Each column of the matrix represents the instances in a predicted class, whereas each row represents the instances in an actual class. The sum of each column represents the number of instances that the classifier predicts, whereas the sum of each row represents the number of instances. All correct guesses are located in the diagonal shape of the confusion matrix.

#### 2.4.2. Classification Accuracy

The classification accuracy rate (true positive rate, TPR) is commonly used as a measurement of the classification results performance. The TPR is calculated as follows:

(12) $$\mathrm{TPR}=\frac{\mathrm{TP}}{\mathrm{TP}+\mathrm{FN}}\times 100\%,$$

where TP represents the correctly identified pollutants, and FN represents the pollutants incorrectly identified as other classes. The higher TPR value is calculated, the better classification performance.

## 3. Experimental and Results

### 3.1. Experimental Data Acquisition

#### 3.1.1. Experimental Design

**Figure 3:**
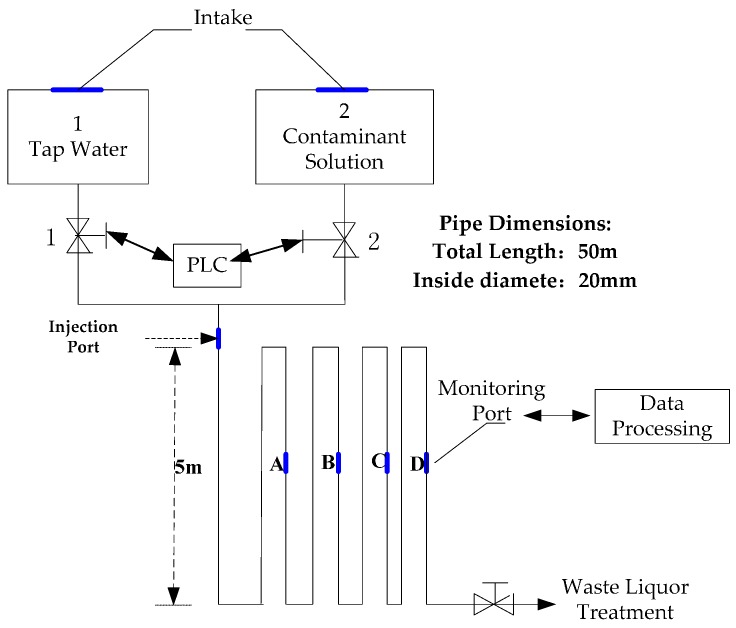
Online water quality monitoring platform.

In Figure 3, a small water distribution system was prepared to simulate the online detection of water quality. The experimental water system includes a storage tank, solenoid valve, pipeline and controller. This system contains a ductile iron pipe for water distribution, which is 50 m long with a 20-mm internal diameter. The on-line monitoring devices were deployed at the four monitoring points on the pipeline according to the distance between the position of the contaminant injection points; subsequently, the online water quality was measured.

Two contaminant injection methods were established: one is the injection with a contaminant solution tank, and the other is the injection through a contaminant injection point. The first injection method was used to investigate the minimum exceptional strength that the system could detect. Although the second injection method was readily apparent to operate, the tap water and the contaminant solution mixing ratio was set closely to the real intentional contamination event through a computer, which was used to investigate whether the system could detect an abnormal pollutant and analyze the classification of the pollutants in real events. The second injection method was adopted in this study.

When contaminants were injected, the target contaminant solution was extracted to a pipeline that was connected to the water tank and sensor by a peristaltic pump. The PLC-controlled flow rate for the extraction of the contaminant solution from the peristaltic pump was determined according to the mixing ratio of the current flow of tap water, mixing rate of the contaminant, and the tap water set in a computer. The mixing proportion was set at 2%. In the classification modeling, the real value of the concentration of contaminant was calculated by mixing the proportion of the concentrates of the contaminants in the initial configuration and tap water. The contaminant solution mixed with tap water was then run through the sensor and subsequently allowed to flow directly into the specified sewage treatment pool.

Before injecting the contaminants, the experimental system remained running to establish a baseline. The sensor data were continually collected and archived automatically to record the sensor response to the injected contaminants. The data were sampled every 1 min. After constructing the baseline, certain concentrations of the contaminant solution were injected. Then, tap water was redirected through the experimental pipe, which permitted the sensor response to return to the baseline levels. The various concentrations of the contaminant solution were injected after the sensor response returned back to the baseline level.

#### 3.1.2. Investigated Contaminants

Three classes and five types of the most common pollutants were selected in the experiment, including agricultural (ammonium citrate), chemical (potassium acid phthalate, potassium ferricyanide, and sodium nitrite) and heavy metal (copper sulfate) pollutants.

**Figure 4:**
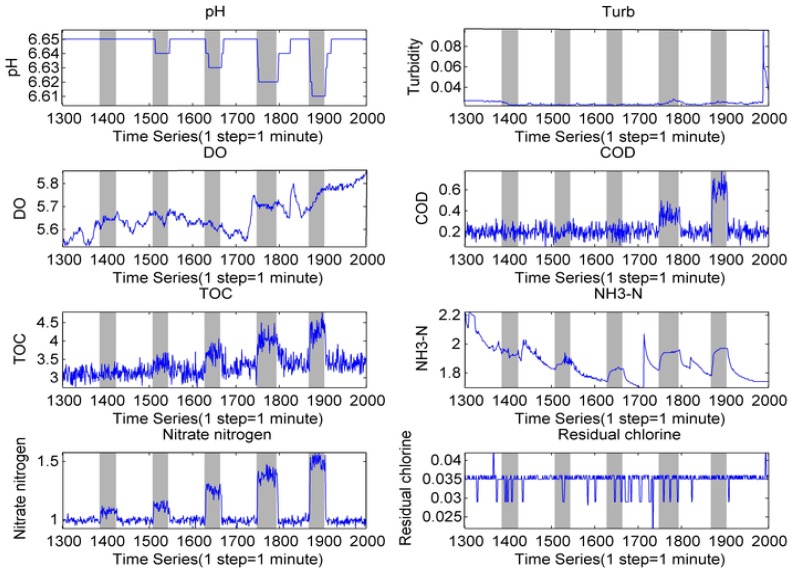
Responses of sensors for potassium acid phthalate.

Figure 4 shows the portion of the data sampled in experiment with potassium acid phthalate as an example. The concentrations of the contaminant solution injected successively equaled to 50, 100, 200, 300, and 400 mg/L, and were run through the sensors corresponding to 1, 2, 4, 6, and 8 mg/L, respectively. In Figure 4, the concentrations of TOC, COD and ammonia nitrogen increased with the injection of potassium acid phthalate, which is related to the sensor response.

In Figure 4 and Appendix A, the indicator is not always identical in response to the different contaminants. Therefore, the contaminants could be classified according to the conventional indicators response. Diverse water quality samples that had an abnormal signal-to-noise ratio were significant to reduce the influence of water quality fluctuations, subsequently improving the accuracy of classification. The residual was selected as a classification characteristic in this study. The residual is the difference between the new observable value and the estimation from the water quality background according to Equation (2). The representation of $Z_{t}$ is the observable value, whereas $Z_{t}^{*}$ is average of the response value before the injection of the contaminants. This average is used as estimation of the water quality background. Residual $\delta_{t}$ is regarded as the relative variance after the injection of contaminants. Herein, the extracting characteristic vectors were obtained by the residuals for every indicator at each time point. In the process of injecting ammonium citrate, the extracting characteristic vectors at each sampling time point were then used to establish a complete feature of the ammonium citrate. In this study, a feature library, including five types of contaminant, was constructed by repeating the above process.

### 3.2. Classification Results of the MCPO Model

#### 3.2.1. Concentration of the Test Pollutant within the Range of Pollutant Library

Five different contaminants were identified, and each of them was injected with five different concentrations for 40 min each. A total of 784 samples were collected. The random selection of a concentration of contaminant in the middle of the concentration gradient (except the minimum and maximum) and the four remaining concentrations of contaminants were regarded as a training set. Thereafter, the total sample set was divided into training and testing sets. The training set comprised 610 samples, whereas the testing set contained 184 samples. The cosine distance classifications were implemented for comparison with the MCPO on the same samples to classify.

Table 1 and Table 2 show the confusion matrix of the two classification methods.

In the confusion matrix, the classification results of the cosine distance classification method and the MCPO method were similar. In two substances, ammonium citrate and potassium acid phthalate, the cosine distance classification method had a slight advantage. For copper sulfate, the classification accuracy of the MCPO method had a larger increase. Table 3 displays the classification accuracy of each material, including the other two distance measurement method. In general, when the concentration of the test pollutant is within range of the pollutant library, the MCPO method has a slight advantage relative to the distance metric.

#### 3.2.2. Concentration of the Test Pollutant outside the Range of Pollutant Library

The same 784 samples were used. The four remaining concentrations of the contaminant were regarded as the training set for the selection of the maximum contaminant concentration. Then, the total sample set was divided into training and testing sets. The training set contained 620 samples, whereas the testing set comprised 174 samples. Table 4 and Table 5 show the confusion matrix of the four classification methods.

In the confusion matrix, the average of the MCPO method had the highest accuracy rate compared with the other three types of methods, reaching approximately 90%. Although the cosine distance method was found to be an efficient distance measurement, its classification accuracy was only approximately 50% in the classification of copper sulfate, which produced serious misclassifications. However the classification accuracy of the MCPO method in copper sulfate reached approximately 80%, which shows significant improvement. Table 6 presents the classification accuracy of each substance using the four methods. When the concentration of the test pollutant was outside the range of the pollutant library, the MCPO method had remarkable advantage relative to the distance metric.

Hence, the classification accuracy of the distance measurement method decreased in entirety when the concentration of the test pollutant was outside the range of the pollutant library, especially in copper sulfate, which is difficult to classify in origin. The classification accuracy was reduced to less than 50%, whereas that of the MCPO method only decreased slightly; thus, the overall difference was not evident.

## 4. Discussion

### 4.1. MCPO Model for Alleviating the Influence of Concentration When Constructing the Pollutants Library

The concentration of the unknown components causes inaccurate classification for pollutants. Liu et al. [8] compared the traditional Euclidean distance metric, namely, Mahalanobis distance measurement, and the cosine distance measure to ease the effects of the unknown concentration of the pollutants. However, some defects were still observed in the process of building the pattern library using mean of instance. This study demonstrates these defects from another perspective, in which the support vector is used through the SVM model to manage the effect of using the mean of instance in the process of building a pattern library.

The decision boundary of the SVM classification model is only decided by a few support vectors. In this approach, only a small number of apparent characteristic samples that can be used in building the pollutant library is required to be extracted. This small number reduces the impact of different concentrations in the process of building the pollutant library. Table 7 presents the support vector constitution of the SVM model obtained by the training set in this study. For example, ammonium citrate has 38 support vectors of the 47 training samples of ammonium citrate (1 mg/L) while only having 10 support vectors out of the 40 training samples (2 mg/L). The high concentration and the few support vector training samples (4 mg/L and 8 mg/L, respectively) contain only 10%. Thus, reducing the influence of concentration and improving the classification precision by focusing on samples with a low concentration and prominent feature when constructing a MCPO model is desirable.

### 4.2. Analysis on Misclassifying the Contaminant Introductionin in the Initial Phase

**Figure 5:**
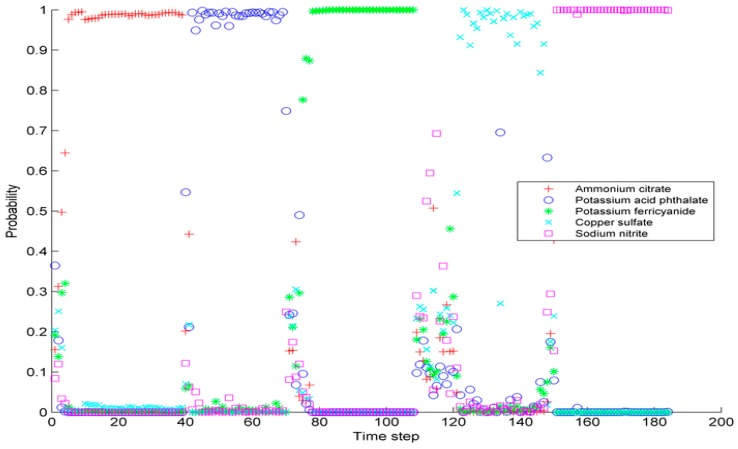
MCPO classification probability.

In the experiment of concentrations of test pollutants within the range of the pollutant library, the classification probability of the 184 test samples via the MCPO method is presented in Figure 5. In this figure, each color represents one classification probability of the contaminant substance from the introduction to termination. In the initial phase of contaminant introduction, the output value of the probability is relatively average and low, which indicates no evidence in determining which type of contaminant and misclassification are likely to occur if single decision is adopted [7].

Table 8 lists the classification probability of the 19 test samples via the MCPO method. To better describe the significance of introducing the classification probability, the sample with the largest real-class classification probability, is denoted as Type I, the second as Type II, the third as Type III, etc. For example, the real class of Sample 4 is potassium biphthalate, and the probability is 21% in the classification probability obtained from the MCPO method. Hence, Sample 4 is denoted as Type III for the third largest output probability. Among the 184 test samples, 167, 10, 6 and 1 Type I, II, III, and IV samples are classified, respectively. The precision of the MCPO would be 90% considering the class with the largest probability as the real class.

The classification probabilities of samples A, B, C, D, and E are denoted p1, p2, p3, p4, and p5, respectively. The maximum probability $P_{\max}$ = max{p1, p2, p3, p4, p5} is determined, and the standard deviations σ of p1, p2, p3, p4, and p5 are calculated. Table 8 presents the statistical result of the samples under Types I, II, III, and IV. Clearly, the mean value of $P_{\max}$ and σ in Sample Set I is large, whereas those of II and III have decreased in order.

For example, Sample 2 falls under Type I, where the maximum classification class is ammonium citrate; the classification probability of this type is 98%; and its σ is 0.436. Furthermore, the classification feature of this type is distinct. Sample 8 falls under Type II, where the maximum classification class, namely, potassium biphthalate, has a probability of 49%, and the real class, namely, potassium ferricyanide, has a probability of 30% and σ of 0.1927. Hence, the classification feature is not considered as distinct.

From the above analysis, the $P_{\max}$ of the classification probability via the MCPO method is large when the classification feature and the standard deviation σ are distinct, and vice versa. The statistical classification results in Table 9 show this result. Hence, using the probability output of this method is desirable in solving $P_{\max}$ and σ to distinguish the samples with distinct or indistinct classification features effectively.

**Figure 6:**
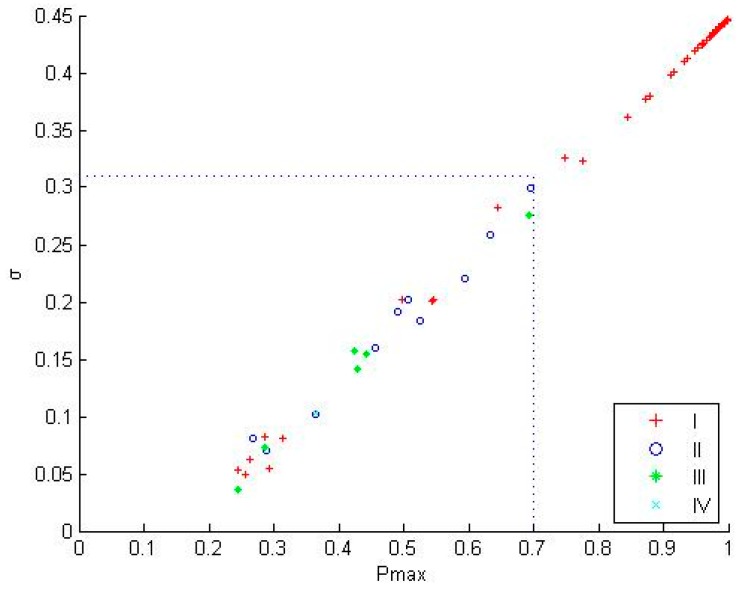
Scatter diagram of the classification.

Figure 6 presents the scatter diagram using the MCPO to classify a test set with 184 samples. Based on the previous analysis, most of the 184 test samples have distinct classification features.

Figure 6 clearly displays that Type I samples have a higher maximum probability and a larger standard deviation, whereas Type II, III, and IV have a lower maximum probability and a smaller standard deviation, i.e., $P_{\max}<0.7$ and σ<0.3, respectively. Collectively, when the classification maximum probability exceeds 0.7, all real classes are the classification probability of the maximum class. The ten real classes of the 27 samples having a maximum classification probability are lower than 0.7, which is similar to the classification probability for the maximum class.

Thus, the samples with large maximum probability and standard deviation have a distinct classification feature, and provide a highly credible single-classification result. However, in the initial phase of the contaminant injection, the classification feature is not as apparent in the samples with small a maximum probability and standard deviation. The single-classification result obtained from conventional method can be necessarily biased. Therefore, in practical use, this method can be utilized to obtain the maximum probability $P_{\max}$ and standard deviation σ in determining whether the sample classification feature is distinct. The criteria provided in this study are $P_{\max}=\;0.7$ and σ = 0.3; that is, samples with $P_{\max}<0.7$, and σ<0.3 do not have distinct classification features. In Figure 6, these samples are concentrated in the initial phase of contaminant injection when the accurate classification result cannot be obtained only from the response data. Thus, the judgment can be considered suspended to avoid an incorrect pronouncement.

## 5. Conclusions

Based on the conventional parameters of water quality and the above analysis, this study presents the on-line classification method of contaminants, and the following conclusions are drawn.

First, on the basis of multi-classification, the online classification method of contaminants can effectively reduce the influence of concentration in constructing a pollutant library, adapt to various substance classifications without a distinct misclassification of certain substance, and significantly raise the classification precision compared with the distance metric classification.

Second, the decision boundary of the MCPO is decided by the support vector, which is primarily composed of low-concentration samples. High-concentration samples can strongly reflect the feature of a certain substance, but insubstantially affect the building of a classification decision boundary.

Finally, the maximum classification probability and its standard deviation can be used to distinguish the samples with distinct or indistinct features. These indistinct samples can appear in the initial injection phase of a contaminant. Hence, a decision cannot be made in this phase when the distinction of classification features are is insufficient.

## Figures and Tables

**Table 1:** Classification results of cosine distance.

	Predict Class	Ammonium Citrate	Potassium Acid Phthalate	Potassium Ferricyanide	Copper Sulfate	Sodium Nitrite
Real Class						
Ammonium citrate		38	0	0	1	0
Potassium acid phthalate		0	29	0	1	1
potassium ferricyanide		1	0	29	8	0
copper sulfate		3	4	1	19	12
sodium nitrite		1	5	0	0	31

**Table 2:** Classification results of MCPO.

	Predict Class	Ammonium Citrate	Potassium Acid Phthalate	Potassium Ferricyanide	Copper Sulfate	Sodium Nitrite
Real Class						
Ammonium citrate		37	0	0	2	0
Potassium acid phthalate		0	27	0	1	3
potassium ferricyanide		0	1	34	3	0
copper sulfate		0	2	0	32	5
sodium nitrite		0	1	0	2	34

**Table 3:** Accuracies of the four methods.

	Classification Method	Euclidean Distance	Mahalanobis Distance	Cosine Distance	MCPO
Test Pollutants					
Ammonium citrate		0.89	0.87	0.97	0.95
Potassium acid phthalate		0.77	0.45	0.93	0.88
Potassium ferricyanide		0.73	0.65	0.76	0.90
Copper sulfate		0.28	0.23	0.48	0.82
Sodium nitrite		0.86	0.81	0.83	0.92
Average		0.69	0.61	0.80	0.90

**Table 4:** Classification results of cosine distance.

	Predict Class	Ammonium Citrate	Potassium Acid Phthalate	Potassium Ferricyanide	Copper Sulfate	Sodium Nitrite
Real Class						
Ammonium citrate		26	0	0	11	0
Potassium acid phthalate		0	22	0	4	3
potassium ferricyanide		2	0	24	10	0
copper sulfate		3	4	3	15	12
sodium nitrite		3	6	0	0	27

**Table 5:** Classification results of MCPO.

	Predict Class	Ammonium Citrate	Potassium Acid Phthalate	Potassium Ferricyanide	Copper Sulfate	Sodium Nitrite
Real Class						
Ammonium citrate		33	0	0	4	0
Potassium acid phthalate		0	25	0	1	3
potassium ferricyanide		0	2	30	4	0
copper sulfate		0	2	0	30	5
sodium nitrite		0	2	0	2	31

**Table 6:** Accuracies of the four methods.

	Classification Method	Euclidean Distance	Mahalanobis Distance	Cosine Distance	MCPO
Test Pollutants					
Ammonium citrate		0.62	0.68	0.70	0.89
Potassium acid phthalate		0.63	0.52	0.75	0.86
Potassium ferricyanide		0.66	0.62	0.67	0.83
Copper sulfate		0.25	0.21	0.40	0.81
Sodium nitrite		0.82	0.72	0.75	0.89
Average		0.60	0.55	0.65	0.86

**Table 7:** Support vector category table.

Contaminant Category		Sample Number	Support Vector Number
Ammonium citrate	1 mg/L	47	38
	2 mg/L	40	10
	4 mg/L	37	3
	8 mg/L	50	6
	Total	174	57
Potassium acid phthalate	1 mg/L	39	29
	2 mg/L	37	29
	4 mg/L	37	18
	8 mg/L	37	6
	Total	150	82
Sodium nitrite	1 mg/L	38	29
	2 mg/L	38	14
	4 mg/L	38	6
	8 mg/L	37	3
	Total	154	52
Potassium ferricyanide	1 mg/L	37	35
	2 mg/L	38	23
	6 mg/L	38	5
	8 mg/L	21	7
	Total	134	70
Copper sulfate	1 mg/L	40	29
	2 mg/L	38	14
	6 mg/L	38	6
	8 mg/L	46	3
	Total	162	52
Total		774	313

**Table 8:** Contaminant classification probability from MCPO.

Sample No.	Contaminant Classification Result						Real Contaminant	SVM Predicted Class
	A	B	C	D	E	Type		
1	0.16	0.36	0.19	0.20	0.08	IV	A	B
2	0.98	0.00	0.01	0.01	0.00	I	A	A
3	0.99	0.00	0.01	0.01	0.00	I	A	A
4	0.44	0.21	0.07	0.22	0.06	III	B	A
5	0.00	0.95	0.00	0.00	0.05	I	B	B
6	0.15	0.25	0.21	0.21	0.17	III	C	B
7	0.42	0.07	0.11	0.31	0.09	III	C	A
8	0.04	0.49	0.30	0.05	0.12	II	C	B
9	0.07	0.01	0.87	0.03	0.02	I	C	C
10	0.20	0.10	0.18	0.23	0.29	II	D	E
11	0.51	0.04	0.09	0.30	0.06	II	D	A
12	0.06	0.07	0.10	0.08	0.69	III	D	E
13	0.27	0.07	0.23	0.26	0.18	II	D	A
14	0.15	0.11	0.46	0.24	0.05	II	D	C
15	0.00	0.04	0.01	0.93	0.01	I	D	D
16	0.00	0.70	0.01	0.27	0.02	II	D	B
17	0.03	0.63	0.07	0.02	0.25	II	E	B
18	0.43	0.08	0.10	0.24	0.15	III	E	A
19	0.00	0.00	0.00	0.00	1.00	I	E	E

Note: A: ammonium citrate; B: potassium biphthalate; C: potassium ferricyanide; D: copper sulfate; E: sodium nitrite; I real type as the largest output probability; II real type as the second largest; III real type as the third largest; IV real type as the fourth largest, etc.

**Table 9:** Statistical classification results.

Type	Quantity	P_max_ Average/%	σ Average
I	167	0.9488	0.42
II	10	0.482	0.1767
III	6	0.4199	0.1398
IV	1	0.3648	0.1033

**Figure A1:**
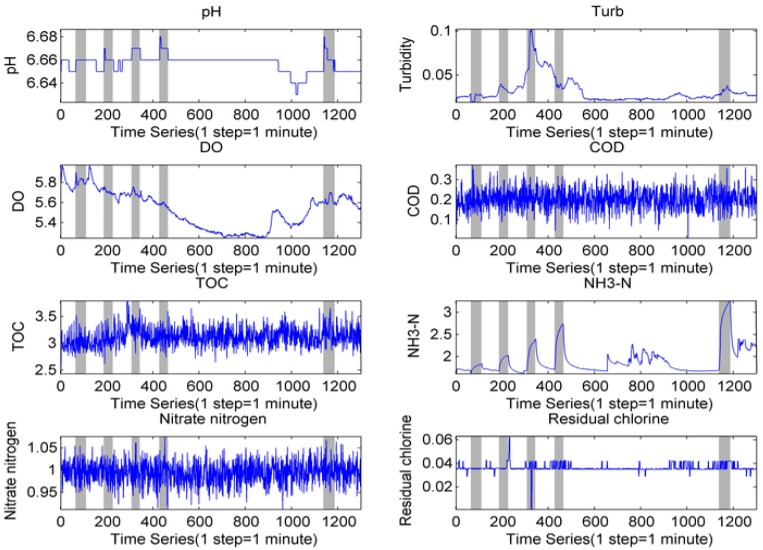
Sensors’ responses for ammonium citrate.

**Figure A2:**
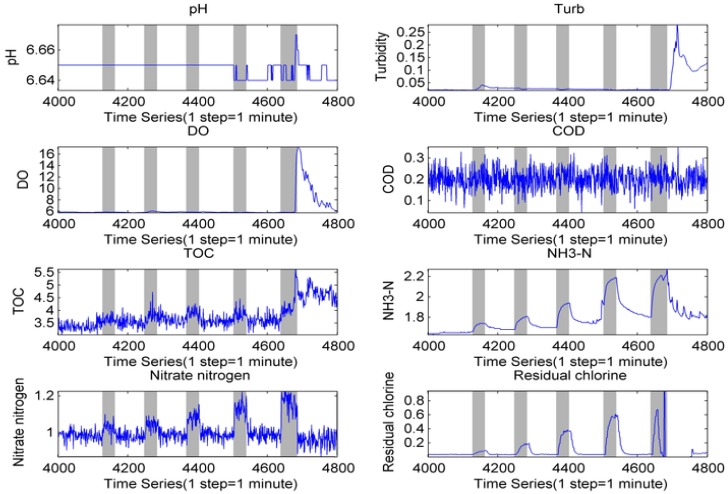
Sensors’ responses for potassium ferricyanide.

**Figure A3:**
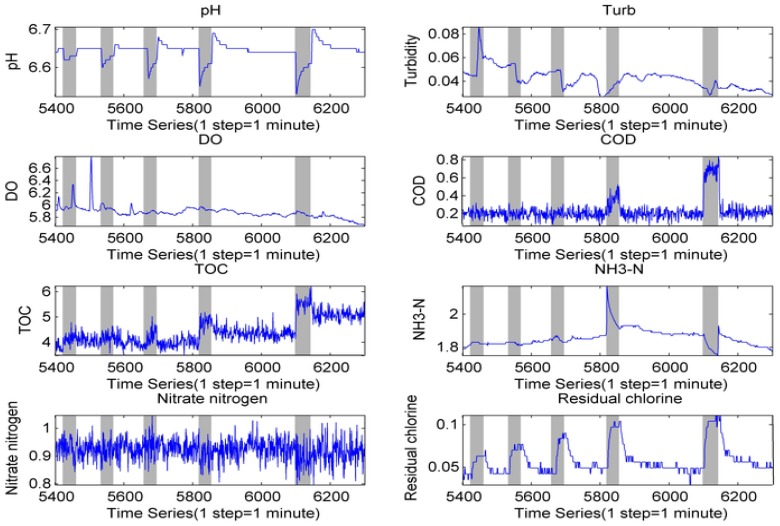
Sensors’ responses for copper sulfate.

**Figure A4:**
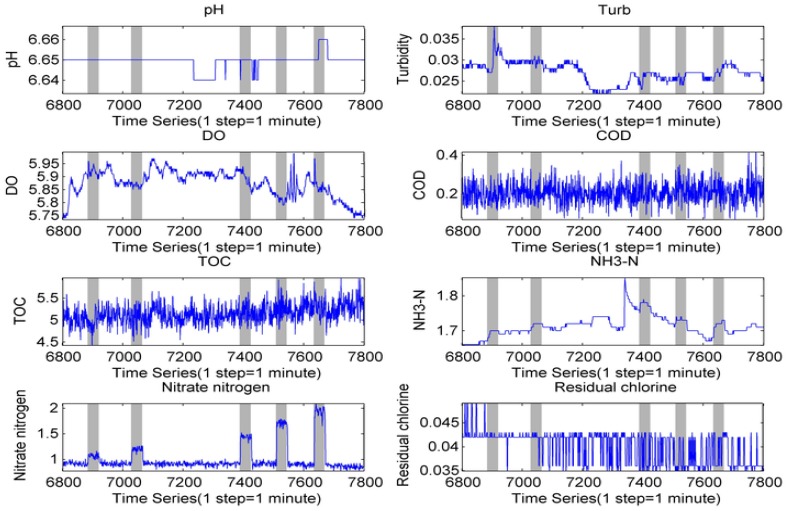
Sensors’ responses for sodium nitrite.
